# Dimension Tailoring of Quasi-2D Perovskite Films Based on Atmosphere Control Toward Enhanced Amplified Spontaneous Emission

**DOI:** 10.3390/ma18194628

**Published:** 2025-10-07

**Authors:** Zijia Wang, Xuexuan Huang, Zixuan Song, Chiyu Guo, Liang Tao, Shibo Wei, Ke Ren, Yuze Wu, Xuejiao Sun, Chenghao Bi

**Affiliations:** 1 College of Physics and Optoelectronic Engineering, Harbin Engineering University, Harbin 150001, China; zijiawang@hrbeu.edu.cn (Z.W.); xuexuanhuang@hrbeu.edu.cn (X.H.); zixuansong@hrbeu.edu.cn (Z.S.); chiyuguo@hrbeu.edu.cn (C.G.); liangtao@hrbeu.edu.cn (L.T.); shibowei@hrbeu.edu.cn (S.W.); keren@hrbeu.edu.cn (K.R.); yuzewu@hrbeu.edu.cn (Y.W.); 2 Qingdao Innovation and Development Center of Harbin Engineering University, Harbin Engineering University, Qingdao 266500, China; 3 Research and Development Center for Wide Bandgap Semiconductors, Institute of Semiconductors, Chinese Academy of Sciences, Beijing 100083, China; xjsun@semi.ac.cn; 4 Yantai Research Institute, Harbin Engineering University, Yantai 264000, China; 5 Sanya Nanhai Innovation and Development Base of Harbin Engineering University, Harbin Engineering University, Sanya 572024, China

**Keywords:** quasi-2D perovskite films, atmosphere control, annealing-free, anti-solvent-free, gain media

## Abstract

Quasi-two-dimensional (Q2D) perovskite films have garnered significant attention as novel gain media for lasers due to their tunable bandgap, narrow linewidth, and solution processability. Q2D perovskites endowed with intrinsic quantum well structures demonstrate remarkable potential as gain media for cost-effective miniaturized lasers, owing to their superior ambient stability and enhanced photon confinement capabilities. However, the mixed-phase distribution within Q2D films constitutes a critical determinant of their optical properties, exhibiting pronounced sensitivity to specific fabrication protocols and processing parameters, including annealing temperature, duration, antisolvent volume, injection timing, and dosing rate. These factors frequently lead to broad phase distribution in Q2D perovskite films, thereby inducing incomplete exciton energy transfer and multiple emission peaks, while simultaneously making the fabrication processes intricate and reducing reproducibility. Here, we report a novel annealing-free and antisolvent-free method for the preparation of Q2D perovskite films fabricated in ambient atmosphere. By constructing a tailored mixed-solvent vapor atmosphere and systematically investigating its regulatory effects on the nucleation and growth processes of film via in situ photoluminescence spectra, we successfully achieved the fabrication of Q2D perovskite films with large *n* narrow phase distribution characteristics. Due to the reduced content of small *n* domains, the incomplete energy transfer from small *n* to large *n* phases and the carriers’ accumulation in small *n* can be greatly suppressed, thereby suppressing the trap-assistant nonradiative recombination and Auger recombination. Ultimately, the Q2D perovskite film showed a single emission peak at 519 nm with the narrow full width at half maximum (FWHM) of 21.5 nm and high photoluminescence quantum yield (PLQY) of 83%. And based on the optimized Q2D film, we achieved an amplified spontaneous emission (ASE) with a low threshold of 29 μJ·cm^−2^, which was approximately 60% lower than the 69 μJ·cm^−2^ of the control film.

## 1. Introduction

Quasi-two-dimensional (Q2D) perovskite films have garnered immense attention due to their exceptional optical properties, including facile solution processing methods, narrow emission bandwidth, unique quantum well structures (QWs), and rapid energy transfer processes, which have emerged as advantageous materials for the next generation of laser gain media [1,2,3]. In general, Q2D perovskite films are formed by introducing large organic cations into precursor solution, resulting in a layered perovskite microstructure with the general formula A_2_-A’_n−1_B_n_X_3n+1_ during the spin coating process. The thickness of the [BX_6_]^4−^ layer between two organic cation layers determines the energy of the quantum wells, denoted here as <n>. Energy and carrier transfer occurs within these QWs, transitioning from high-energy smaller <n> phases to low-energy larger <n> phases for recombination and luminescence [4]. During this energy transfer process, photogenerated carriers are induced to concentrate at larger <n> phases, significantly aiding in achieving population inversion [5], which holds promise for achieving lower laser thresholds. Furthermore, the QWs can effectively confine the excitons and reduce the exciton diffusion lengths, which can decrease the probability of exciton–defect quenching [3,4]. It is found that the small <n> phase Q2D perovskite films are not suitable for lasers due to their strong electron–phonon interaction and faster band edge-to-trap process and the lack of the optical gain of double excitons [4,6,7]. In addition, compared with the small <n> phase Q2D perovskite films, the exciton binding energy of the photoinduced carriers concentrated in the large <n> phase Q2D perovskite is smaller, and the Auger recombination that limits the laser threshold is weaker. Research shows that the large <n> Q2D perovskite films with a narrow phase distribution are much preferred to promote carrier transport and reduce extra energy loss for achieving low lasing thresholds [4,5]. Therefore, a reasonable and uniform phase distribution is very important to reduce the lasing threshold. The main factors influencing the distribution of <n> values are the nucleation and growth processes. Therefore, it is important to focus on the nucleation and growth process of Q2D perovskite films. By regulating this process, a reasonable phase distribution and reduced defect density of the films can be achieved, thereby facilitating rapid energy transfer and lowering the lasing threshold.

The accurate manipulation of processing parameters is of paramount importance for regulating the film growth and nucleation process to obtain high-quality Q2D perovskite films with narrow phase distribution and efficient energy transfer [8]. However, the majority of researchers currently employ antisolvent and annealing methods for their fabrication processes, which introduces numerous influencing factors, including annealing temperature, annealing duration, the timing of antisolvent addition, and the rate of antisolvent addition, leading to increased batch-to-batch variation and reduced reproducibility. Xiao et al. conducted an investigation of the annealing temperature and proposed that both higher and lower annealing temperatures would degrade the performance of the perovskite films [9]. Additionally, it has been proved that the emission wavelength difference of Q2D perovskite films is influenced by annealing temperature [10], which shows the influence of quantum confinement relative to the particle size of nanograins [11]. Furthermore, there will be charged vacancies at the surface and grain boundaries during high-temperature annealing [12], which leads to an increase in the lasing threshold. Regarding the antisolvent method, previous studies have demonstrated a strong correlation between the antisolvent dripping time and the resulting film morphology. For example, Alexander et al. studied the effect of 14 different antisolvents on the formation of Q2D perovskite films; their research showed that by changing the duration of antisolvent application (the time of antisolvent addition), even the same antisolvent can produce devices with different efficiencies, and the optimal application duration of different antisolvents is not the same [13]. Furthermore, factors such as antisolvent volume, temperature, additive dosage, and spin coating parameters also exert influences on the quality of the films [14,15,16]. However, research on these variables remains incomplete, leading to some contradictory conclusions. In order to ensure reproducibility of high-quality Q2D films, it is crucial to eliminate the impact of the aforementioned variables. This holds significant importance for the laser application of Q2D perovskite films. For this reason, it is necessary to develop a straightforward method that facilitates rapid nucleation and growth of Q2D perovskite films, leading to the achievement of a low lasing threshold. The environmental atmosphere is critical for the preparation of high-quality Q2D perovskite films. For example, the solvent vapor annealing (SVA) method commonly used today involves placing the material together with an organic solvent on a temperature-controlled stage in a closed environment. At a given annealing temperature, the organic solvent evaporates and gradually diffuses into the material, driving film crystallization and influencing film morphology [17]. In the study of the effect of DMSO atmosphere on the nucleation and growth process of Q2D perovskite films, Jin et al. also proposed that the partial pressure of the solvent is a key factor controlling the in situ crystal of perovskite, and that the solvent vapor atmosphere significantly affects the nucleation and growth of perovskite crystals [18]. Furthermore, recent research on the reconstruction of passivation defects in Q2D perovskite crystals assisted by water molecules indicates that water molecules in the ambient atmosphere do indeed have an impact on the alignment of Q2D perovskite lattices [19]. All the above studies suggest that environmental molecules in the atmosphere can have a certain influence on the manufacturing of Q2D perovskite films.

In this work, we propose a novel method for synthesizing high-quality Q2D perovskite films based on an atmosphere-controlled method. We first precisely construct various environmental atmospheres by volatilizing the saturated vapor of common antisolvents, such as chlorobenzene (CB), toluene (TL), ethyl acetate (EA), or isopropanol (IPA), into the spin coating environment, and then conducting a two-step spin coating process within this atmosphere. Subsequently, we systematically investigated the effects of various solvent vapor atmospheres on the nucleation and growth dynamics of Q2D perovskite films through the integration of multiple in situ monitoring techniques. We found that the atmosphere behavior was similar to that of an antisolvent, which can effectively adjust nucleation and growth of perovskite and remove small *n* phase and trap states. favoring the generation of a narrow distribution of <n> phases and suppression of nonradiative recombination process. Ultimately, based on the optimized composition of solvent vapor, we successfully synthesized dense and narrow-phase-distribution Q2D perovskite films with a single emission peak at 519 nm, a full width at half maximum (FWHM) of approximately 21.5 nm, and a PLQY of 83%. The same Q2D perovskite film produced ASE with a relatively low threshold of 29 μJ·cm^−2^. Our method exhibits the advantages of being annealing-free, antisolvent-free, simple, and convenient, making it suitable for large-scale fabrication of Q2D perovskite films. The resultant films demonstrate high uniformity and excellent batch-to-batch reproducibility.

## 2. Results and Discussions

**Figure 1:**
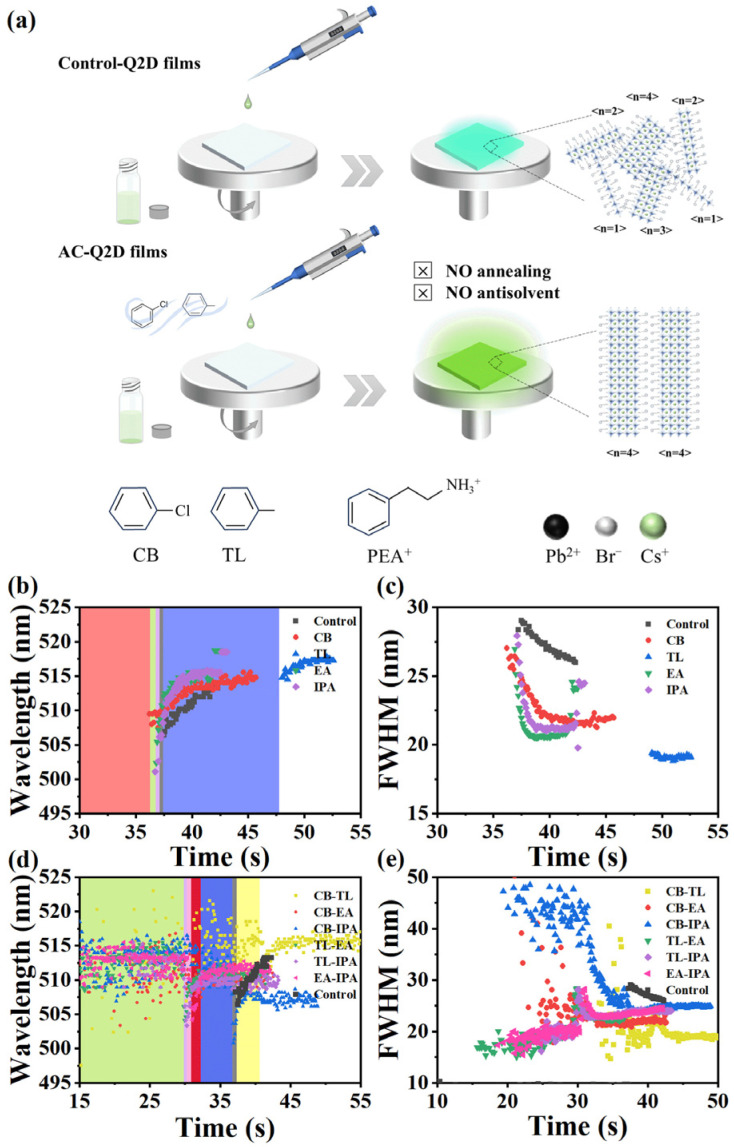
(**a**) Schematic illustration of the atmosphere-controlled method to fabricate the control-Q2D films and AC-Q2D films. The evolution of (**b**) PL emission peak position and (**c**) FWHM of Q2D films by single-atmosphere control; the evolution of (**d**) PL emission peak position and (**e**) FWHM of Q2D films by multiple-atmosphere control.

Conventionally, as shown in Figure 1a, Q2D perovskite films can be readily fabricated by directly spinning precursor solutions onto substrates (named as control-Q2D films). However, the inherently uncontrolled nucleation and growth dynamics typically lead to disordered phase distribution within the resulting films. Although antisolvent dripping during spin coating has been widely adopted to control nucleation and the growth process, the stringent requirements for precise control over processing parameters such as antisolvent volume, injection timing, and solvent evaporation rate often result in compromised process reproducibility. Consequently, based on the underlying principles of antisolvent engineering, we developed a solvent vapor atmosphere-controlled approach utilizing conventional antisolvents to regulate crystallization pathways by spin-coating the precursor solution in a special atmosphere including mixed CB and TL vapor in equal proportions. The Q2D perovskite films can be obtained which are more uniform and denser compared to films produced in a regular atmosphere. This process can even remove thermal annealing or antisolvent treatment. Q2D films obtained by this atmosphere-controlled method in the optimized environment (AC-Q2D films) exhibit narrow and consistent <n> phases and well-ordered arrangements, with significant improvements in optical performance and stability across various aspects. Also, the batch-to-batch reproductivity can also be significantly improved due to the simplified process. For specific experimental details, please refer to the Experimental Section in the Supplementary Materials.

As illustrated in Figure 1b–e, an in situ photoluminescence (PL) spectra system is carried out to monitor and reveal the nucleation and growth mechanism of Q2D films under various solvent atmospheres. Initially, the effects of the conventional antisolvent of chlorobenzene (CB), toluene (TL), ethyl acetate (EA), and isopropanol (IPA) as an atmosphere on the nucleation and growth of Q2D perovskite films are analyzed in Figure S1a–e. Compared to the control-Q2D films, the introduction of CB, EA, and IPA can enhance the formation rate of Q2D films. Although the nucleation time is approximately similar to that of the control-Q2D film, the growth rate is significantly increased (reduced from 11.6 s to within 3 s). By observing the changes in the PL emission peak position during the nucleation and growth of the films, it is observed that all films prepared by the introduction of these atmospheres show the gradually larger grain size and <n> phase during spin coating, accompanied by a redshift of the PL emission peak [18]. Compared to the control-Q2D films, the films prepared under the EA atmosphere show that the largest redshift occurred, indicating that EA atmosphere can efficiently eliminate the smaller <n> phase. Additionally, we extracted the changes in the FWHM of the films, as shown in Figure 1c. It is evident that the FWHM of the films decreased by varying degrees compared to the control-Q2D films after the introduction of solvent atmospheres, suggesting a gradually uniform size and phase distribution. Notably, the film prepared in TL showed the highest decrease in the FWHM from 26.0 nm to 19.1 nm, indicating that TL atmosphere is beneficial for uniform phase distribution. Films treated with a single atmosphere exhibited performance enhancement. However, the overall performance remained less than ideal. Therefore, in order to achieve the synergistic effect of different solvent atmospheres, we designed the mixed-solvent atmosphere based on the above two atmospheres together and studied the effects on the nucleation and growth of Q2D films, as shown in Figure 1d,e and Figure S1f–k. After mixing, the advantages of individual atmospheres are more superimposed, but the optimization effect of the mixed atmosphere is closely related to whether the boiling points of the antisolvent are similar. As a result, due to the similar boiling points of CB and TL, CB-TL shows the faster nucleation rate and the longer PL emission peak wavelength, indicating a larger size of the grains and <n> phase than other atmospheres. Meanwhile, the uniformity of its internal phase distribution is well maintained, with an FWHM of only 19 nm. Based on the above analysis, we ultimately selected CB and TL, which exhibited the most advantages, to constitute the atmospheric environment in equal proportions for the preparation of AC-Q2D films. In addition, we tested the batch-to-batch variation and reproducibility of AC-Q2D films through a large number of experiments and statistical data. Figure S2 shows that the peak position movement range of PL in the control-Q2D films is approximately 510–526 nm, the variation range of FWNM is mostly concentrated around 20–25 nm, with a few cases being between 10 and 20 nm and 25 and 50 nm, and the variation range of PLQY is approximately between 10% and 32%, with very few cases being lower than 10% or higher than 32%. The peak position movement range of PL in AC-Q2D films is between 517 nm and 522 nm, the variation range of FWNM is between 20 nm and 24 nm, and the variation range of PLQY is between 75% and 83%. Compared with control-Q2D films, the PL peak position movement range, the FWNM variation range, and the PLQY variation range of AC-Q2D films are all smaller. Therefore, the reproducibility of AC-Q2D films is significantly improved, and the batch-to-batch variation is also significantly enhanced.

**Figure 2:**
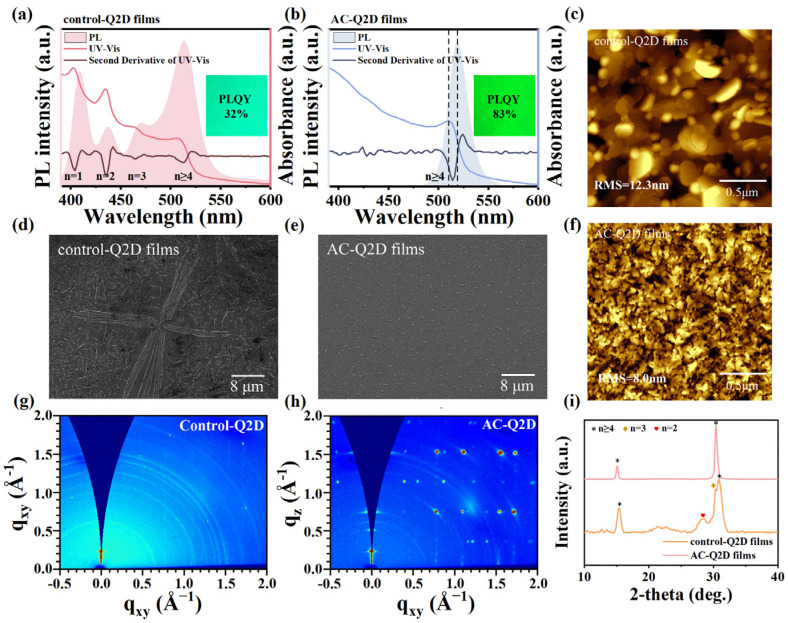
Normalized PL absorption spectra along with the second-order derivative analysis derived from the absorption spectra for (**a**) control-Q2D films and (**b**) AC-Q2D films. Electron scanning electron microscopy (SEM) images of the (**d**) control-Q2D films and (**e**) AC-Q2D films. Atomic force microscopy (AFM) images of the (**c**) control-Q2D film and (**f**) AC-Q2D film. GIWAXS patterns of (**g**) control-Q2D films and (**h**) AC-Q2D films. (**i**) XRD patterns of the control-Q2D and AC-Q2D films.

The normalized PL spectra and ultraviolet–visible absorption spectra (UV-vis) of the control-Q2D films and AC-Q2D films are displayed, respectively, in Figure 2a,b. In order to visually identify the absorption peaks in the absorption spectra, we performed a second-order derivative analysis on the absorption spectra. The multiple excitonic absorption peaks of the control-Q2D films, including *n* = 1 (402.0 nm), *n* = 2 (436.1 nm), *n* = 3 (465.5 nm), and *n* ≥ 4 (514.8 nm), can be identified in the second derivative of absorption spectra, suggesting that the film features the QW structure with different bandgaps [3]. The multiple and broadened PL emission peaks of the control-Q2D films can be attributed to the incomplete internal energy transfer due to the coexistence of multiple phases. Radiative recombination occurs within each quantum well, leading to complex luminescent properties. In contrast, the observed pure green emission of the AC-Q2D films can be attributed to a reasonable distribution of <n> phases. The AC-Q2D films show a narrower and single PL emission peak at 519 nm with an FWHM of 22.4 nm, and they also show the single excitonic absorption peak (*n* ≥ 4, 510 nm), which indicates the concentrated and narrow phase distribution in the AC-Q2D films. In addition, the AC-Q2D films exhibit a slight redshift of the dominant PL emission peak compared with the control-Q2D films, which was attributed to weaker quantum confinement [20], confirming the higher ratio of larger <n> phases in the AC-Q2D films. The AC-Q2D films also show a larger Stokes shift (≈42 meV (≈9 nm)) resulting from a confined hole state above the valence band edge [21] than that of the control-Q2D films, which is beneficial for reducing the self-absorption of emitted photons and further lowering the ASE threshold [22]. Importantly, the photoluminescence quantum yield (PLQY) of the AC-Q2D films (83%) is much higher than that of the control-Q2D films (32%). This indicates that the trap states inside the AC-Q2D films were efficiently eliminated and undesired nonradiative recombination was reduced [23]. Also, the narrow larger <n> phase distribution accelerated excitons transfer process contributes to the highly efficient radiative recombination [3]. The conclusion is also consistent with the results of the scanning electron microscope (SEM) and atomic force microscope (AFM). As shown in Figure 2d–f, the control-Q2D films exhibit rough surface and large pin-like grains, and the root mean square (RMS) of the films is large, approximately 12.3 nm. Instead, in the AC-Q2D films, the large pin-like grains nearly disappear; the AC-Q2D films show the small, round grains and compact and smooth surface, and the RMS is reduced to 8.0 nm. It can also be seen from the SEM cross-sectional view of the film in Figure S3 that the AC-Q2D films are more uniform and denser, while the control-Q2D films have more large grains and are relatively rough. Moreover, the thicknesses of the control-Q2D films and the AC-Q2D films are approximately 67 nm and 60 nm, respectively. The results demonstrate that the solvent atmosphere facilitates the formation of abundant nucleation sites, which promotes uniform and compact growth of Q2D perovskite crystals while simultaneously narrowing their phase distribution. Figure 2i shows a typical X-ray diffraction (XRD) pattern obtained from the control-Q2D films and AC-Q2D films. The diffraction peaks at 15.4°, 21.5°, 28.4°, 30.4° and 30.9°, of the control-Q2D films can be attributed to the complex phase distribution [24], which is consistent with the results of the PL and UV spectra. In the AC-Q2D films, the diffraction peaks at 21.5°, 28.4°, and 30.9° disappear, and only two diffraction peaks at 15.4° and 30.4° can be observed, which shows that the small *n* phase decreased and the phase distribution was more uniform.

The high crystallinity and vertical orientation of the AC-Q2D films are further confirmed by synchrotron grazing-incidence wide-angle X-ray scattering (GIWAXS) measurements [25], where distinct Bragg spot features are observed predominantly in the out-of-plane (q_z_) direction (see Figure 2g). As shown in Figure 2h, the diffraction rings presented in the GIWAXS pattern of the control-Q2D films suggest that in the absence of atmosphere controlling, a polycrystalline perovskite film with random orientations is formed. The diffraction rings for the AC-Q2D films became weak and even disappeared, and several diffraction dots can be observed, which suggests the preferential orientations of AC-Q2D films. The sharp and distinct diffraction dots revealed the formation of highly oriented perovskite film, suggesting that the crystals are almost in the same phase distribution and orientation [26]. In the integrated intensity−q relations of GIWAXS patterns (Figure S4), the AC-Q2D films show significant features of multi-order diffraction corresponding to *n* ≥ 4. Several diffraction dots appeared in the small q-value region of the AC-Q2D films, which is located at 0.16 A°^−1^, and the corresponding periodic distance for this peak is 38.96 A°. The result of GIWAXS strengthens our confidence in the validity of the atmosphere-controlled method.

**Figure 3:**
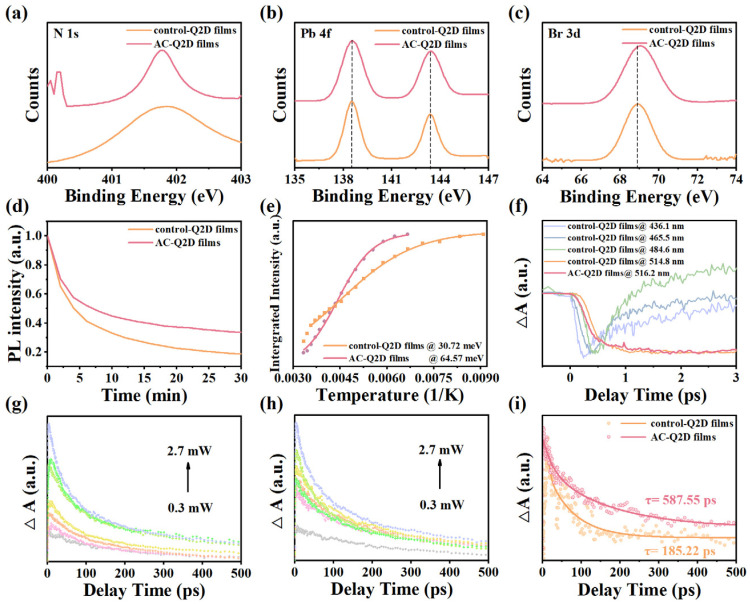
(**a**) XPS N 1s spectra, (**b**) XPS Pb 4f spectra, (**c**) XPS Br 3d spectra of the control-Q2D and AC-Q2D films. (**d**) Effect of storage time on the PL intensity of the control-Q2D and AC-Q2D films. (**e**) The plot showing integrated PL emission intensity as a function of temperature of the control-Q2D and AC-Q2D films. (**f**) Kinetics of the GSB of the control-Q2D and AC-Q2D films. Pump-intensity-dependent transient TA kinetics of (**g**) control-Q2D films and (**h**) AC-Q2D films; (**i**) kinetics of biexciton recombination.

X-ray photoelectron spectroscopy (XPS) measurement is conducted to explore the surface environment of AC-Q2D films. As shown in Figure 3a, the AC-Q2D perovskite films show a narrow signal peak at 401.8 eV, which corresponds to the N 1s spectra of the PEA^+^. In contrast, the broader distribution of N 1s spectra for the control-Q2D films is attributed to the complex state of PEA^+^, leading to phases with different <n> values. In addition, the AC-Q2D films show the decreased integrated area of N 1s spectra, which suggests the reduced ratio of the surface PEA. This observation implies that the majority of the PEA^+^, following atmosphere-controlled optimization, is confined within the film interior, forming a uniform and narrow-distribution Q2D structure. Consequently, the atmosphere-controlled method effectively optimizes the phase distribution within the Q2D perovskite films. As demonstrated in Figure 3b,c, the binding energies corresponding to the Br 3d and Pb 4f core levels of the AC-Q2D films all slightly shift to higher values compared to the control-Q2D films, indicating the stronger coordination between Br and Pb ions on the perovskite surface; this is because of the change in the electron cloud density, indicating that there is an interaction between the solvent atmosphere and precursor and further suggesting that the AC-Q2D film surface could be electrostatically passivated by the atmosphere [27]. As depicted in Figure 3d, the AC-Q2D films show a better stability under continuous illumination by a xenon lamp light source at 365 nm than that of the control-Q2D films, which is attributed to their internally dense grain morphology and lower trap state density.

As shown in Figure S5, the temperature-dependent PL spectra with temperatures from 80 to 300 K for the samples is carried out to study the exciton behaviors within Q2D films. Figure 3e plots the relationship between integrated PL intensity and temperature, and the exciton binding energy was calculated by fitting the plot using Equation (S1) in Supplementary Materials [28]. The exciton binding energy (E_b_) is extracted from the fitting line from Figure 3e; the AC-Q2D films show a much higher E_b_ (~64.57 meV) than that of the control-Q2D films (~30.72 meV), which can be attributed to the reduction in defect states [29]. Hence, we think that the higher PLQY of the AC-Q2D films can be attributed to the efficient exciton recombination due to the increased exciton binding energy [30]. At a temperature of 80 K, the FWHM of the control-Q2D films and AC-Q2D films narrowed to 16.68 nm and 11.98 nm, respectively. This confirms that modifications in the ambient atmosphere have significantly contributed to the reduction in internal defect density and the enhancement of crystalline quality within the films [17]. Furthermore, the central PL peak wavelength of the control-Q2D films shows a continuous blueshift of approximately 9 nm as the temperature increases, which is attributed to the stabilization of out-of-phase band edge states as the lattice expands [31]. In contrast, the central wavelength of the AC-Q2D films remains stable, with almost no observable blueshift with the increasing temperature. The time-resolved PL (TRPL) decay spectra are conducted to investigate the exciton decay dynamics in Q2Ds. As shown in Figure S6 and Table S1, the decay dynamics of both the control-Q2D and AC-Q2D films can be fitted by a bi-exponential decay function [32], and the AC-Q2D films exhibit longer PL lifetimes of τ = 5.67 ns than that of the control-Q2D films (τ = 2.59 ns). The higher PLQY and longer exciton lifetime suggest that the surface defects have been effectively removed in the AC-Q2D films, resulting in the significant suppression of the trap-mediated nonradiative monomolecular recombination [33]. To further understand the photophysical properties of control-Q2D and AC-Q2D films, transient absorption spectroscopy (TAS) is used to further study the specific exciton behaviors. Figure S7 shows the time evolution of the TAS spectra of control-Q2D and AC-Q2D films with ground state bleaching (GSB) signals ranging from 400 to 600 nm. There are multiple GSB signals shown in the control-Q2D films; the observed GSB peaks at 436.1 nm, 465.5 nm, 484.6 nm, and 514.8 nm could be ascribed to the state filling induced by photoexcited excitons in different *n* phases and match well with excitonic absorption peaks in the steady absorption spectra. In contrast, the AC-Q2D films show only a single signal peak at 516.2 nm, indicating the more concentrated phase distribution in AC-Q2D films. To obtain the relationship between the carrier dynamics of control-Q2D films and AC-Q2D films, we extracted the transient dynamics curves at specific wavelengths (514.8 nm, 516.2 nm) and normalized all the curves, as shown in Figure S8. The carrier recombination dynamics data were fitted using a double exponential function. The obtained time components are shown in Table S2. The carrier dynamics lifetimes after fitting by the control-Q2D films and AC-Q2D films were 644.52 ps and 760.29 ps, respectively. Compared with control-Q2D films, AC-Q2D films have a longer carrier dynamic lifetime, further confirming the lower defect state density and fewer nonradiative recombination processes within AC-Q2D films. As shown in Figure 3f, the TA kinetics show that there is complex energy transfer from small *n* to large *n* domains. For the control-Q2D films, the GSB at the large *n* phase shows a fast rise time accompanied by the rapid relaxations and slow decay of the small *n* phase, indicating a fast buildup of excitons in the narrowest bandgap. In contrast, the AC-Q2D films show the negligible decay of the smaller *n* phase and the shorter rising time of the large *n* phase. The faster exciton accumulation time for the large *n* phase in the AC-Q2D perovskite film confirms an accelerated carrier transfer process. Due to the presence of complex phase distributions and energy transfer processes, only a general overview of the processes can be observed. Meanwhile, according to singular value decomposition (SVD) global fitting, the TA dynamics can be decomposed into three components, which are shown in Figure S9. The three components represent the lifetime of hot excitons, the trapping time constant, and the recombination time constant, respectively. For the time-dependent evolution of the TA spectra of the AC-Q2D films, we attribute the PA1 signal within the wavelength range of 460 nm to 500 nm to the absorption induced by the lowest exciton state [34]. The PA2 signal within the wavelength range of 530 nm to 550 nm is generally attributed to a Stark-like effect arising from the Coulomb interaction between hot excitons and band edge excitons [35]. As observed in Figure S9d–f, the PA2 signal is formed at approximately 0.4 ps. Subsequently, the PA2 signal decays alongside the formation of the ground state bleaching signal, a phenomenon attributed to the formation of band edge excitons during the relaxation process of hot carriers [36]. In addition, Auger recombination is another important factor that determines the laser threshold [4]. As shown in Figure 3g,h, we investigated the recombination dynamics of excited excitons probed at the exciton bleach under a series of pump intensities. The rapid growth of a fast-decay component in response to heightened pump intensity aligns with the observed behavior indicative of biexciton recombination. The kinetics of biexciton recombination are derived through a subtractive method [5,37,38] and modeled using single-exponential decay functions for fitting purposes. As depicted in Figure 3i, the time constants of the control-Q2D films and AC-Q2D films are 185.22 ps and 587.55 ps, respectively. This suggests that the Auger recombination in the AC-Q2D films is much weaker than that in the control-Q2D films. These results imply that the atmosphere-controlled method can lead to a narrow phase distribution dominated by large *n* domains in the Q2D film, which benefits the complete energy transfer. Photoinduced carriers can be concentrated on the large *n* phase to reduce the local carrier density of the small *n* phase, thus suppressing the Auger recombination. Based on the aforementioned analyses, the AC-Q2D film exhibits a significantly more comprehensive energy transfer process, with a substantial suppression of Auger recombination occurring [39].

**Figure 4:**
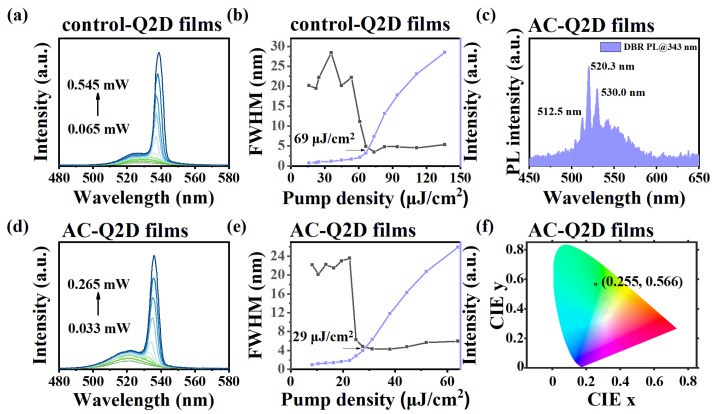
Pump-fluence-dependent PL spectra of (**a**) control-Q2D films and (**b**) AC-Q2D films. PL intensity and FWHM as a function of pump fluence of (**d**) control-Q2D films and (**e**) AC-Q2D films. (**c**) The PL spectrum of AC-Q2D films embedded in DBR. (**f**) The CIE coordinate of AC-Q2D films.

Regarding lasers, the performance hinges significantly on the gain property of the gain media, which exhibits a strong link to the crystallization quality of perovskite crystals and films [40]. In Figure 4a,b,d,e, the amplified spontaneous emission (ASE) characteristics of the control-Q2D films and AC-Q2D films are demonstrated by plotting the PL spectrum as a function of laser pumping fluence and extracting the relationship between the PL intensity or FWHM and pump power density. At a low pumping level, the emission spectra are similar to the above PL spectra (Figure 2b) with a peak at 520 nm with an FWHM of ~22.2 nm for the AC-Q2D films. When the pumping intensity is increased, an intense emission shoulder appears at 536 nm with the FWHM rapidly decreasing to ∼5 nm, corresponding to the generation of ASE. The evolution of PL intensity and FWHM as a function of pump fluence indicates that the threshold of the AC-Q2D films show a significant reduction from 69 μJ/cm^2^ to 29 μJ/cm^2^, representing a decrease of approximately 60%. Moreover, Figure S10 shows the ASE intensity attenuation curves of the AC-Q2D film and the control-Q2D film within 30 min under their respective two-fold threshold excitations. The ASE time stability of the AC-Q2D film is significantly better than that of the control-Q2D film. This is attributed to the uniform phase distribution and dense morphology of the film, which results in an extremely low defect density and complete energy transfer, leading to the suppression of trap-assistant monomolecular nonradiative recombination and Auger recombination through the atmosphere-controlled method. Here, a distributed Bragg reflector (DBR) mirror structure is incorporated into AC-Q2D films, and excitation is performed using a 343 nm femtosecond pulsed laser. Consequently, a multi-peaked PL spectrum was obtained, as shown in Figure 4c. However, we did not obtain a highly coherent single-peak emission laser. This might be due to the thickness mismatch of the Q2D perovskite film, resulting in a mismatch between the cavity length and the target resonant wavelength. As shown in Figure S3, the thickness of the AC-Q2D film is approximately 60 nm. According to the phase matching condition in Equation (S2) in Supplementary Materials [41], under the condition that m is an integer, the resonant wavelength is between 521 and 532 nm, which is not within the ASE wavelength range of the AC-Q2D film. Moreover, since the thickness of the AC-Q2D film hardly changes without altering the experimental conditions, the unregulated film thickness ultimately hinders the generation of highly coherent single-peak emission lasers. Meanwhile, as shown in Figure 4f, the corresponding Commission Internationale de I’Eclairage (CIE) chromaticity coordinates of the AC-Q2D films are (0.255, 0.566), revealing a green emission with high color purity.

## 3. Conclusions

In summary, we propose a method for fabricating high-quality Q2D perovskite films without annealing or antisolvent treatment, based on an atmosphere-controlled method. By analyzing the effects of various antisolvent atmospheres based on in situ PL measurement, we verified the positive effect of the atmosphere-assisted method on the nucleation and growth process of Q2D perovskite films. Based on this, we conducted a comparative experiment on the preparation assisted by a doped atmosphere environment. The results show that atmosphere environment doping needs to meet the prerequisite that the boiling point of the atmosphere combination is close. Eventually, we ultimately construct an optimized atmosphere including equal proportions of CB and TL, effectively suppressing the formation of the small *n* phase, the incomplete energy transfer from the small *n* to large *n* phases and the carriers’ accumulation in small *n* can be inhibited, thereby suppressing the trap-assistant nonradiative recombination and Auger recombination. The resulting AC-Q2D perovskite films exhibit a large *n* narrow phase distribution with a single PL emission peak at 519 nm, a narrow FWHM of 22.4 nm, and a high PLQY of 83%. On the basis of the AC-Q2D films, we achieve a green ASE with approximately 60% lower threshold from 69 to 29 μJ·cm^−2^ than that of the control-Q2D films, representing a substantial performance improvement. But the atmosphere method is currently unable to regulate the thickness of Q2D films and cannot meet the requirements of different film thicknesses. In future research, the precise control of Q2D film thickness and the exploration of phase matching between Q2D films and DBR lasers will play an important role in promoting the development of Q2D perovskite lasers. Despite these limitations, our study provides a new idea for the preparation of Q2D perovskite films with high optical properties and also offers a significant boost for the development of novel lasers.

## Data Availability

The original contributions presented in this study are included in the article. Further inquiries can be directed to the corresponding author.

## Supplementary Materials

The following supporting information can be downloaded at: https://www.mdpi.com/article/10.3390/ma18194628/s1, Supplemental Information, including Supplemental Experimental Procedures, figures, and tables can be found with this article online. Experimental Section: It includes materials, preparation of PEA_2_-Cs_n-1_Pb_n_Br_3n+1_ precursor, preparation of PEA_2_-Cs_n_-_1_Pb_n_Br_3n+1_ Q2D films, characterizations, the ASE Measurements, the Laser Measurements and the equations used in the text; Figure S1: In-situ PL spectra of different solvent atmosphere Q2D films; Figure S2: comparison of batch-to-batch variation and reproducibility between control-Q2D films and AC-Q2D films; Figure S3: cross-sectional SEM images of control-Q2D films and AC-Q2D films; Figure S4: the integrated intensity−q relations of GIWAXS patterns of AC-Q2D films; Figure S5: pseudo color map of temperature-dependent PL spectra of control-Q2D films and AC-Q2D films; Figure S6: TRPL decay and fitting curve of the control-Q2D films and AC-Q2D films; Figure S7: TA spectroscopy and the SVD global fitting results of control-Q2D films and AC-Q2D films; Figure S8: TA dynamics decay and fitting curve of the control-Q2D films and AC-Q2D films; Figure S9: time-dependent evolution of the TA spectrum of control-Q2D films and AC-Q2D films in the different time scales; Figure S10: the ASE time stability of control-Q2D and AC-Q2D films; Table S1: summary of TRPL fitting parameters for control-Q2D films and AC-Q2D films; Table S2: summary of TA dynamics spectra fitting parameters for control-Q2D films and AC-Q2D films.
