# Supplementary material for: Dimension Tailoring of Quasi-2D Perovskite Films Based on Atmosphere Control Toward Enhanced Amplified Spontaneous Emission
Source: Materials (Basel). 2025 Oct 7;18(19):4628. doi: 10.3390/ma18194628 (PMC12525978; doi:10.3390/ma18194628)
Supplement: Supplementary file 1 [file materials-18-04628-s001.zip › materials-3795959-supplementary.pdf]

Supplementary Materials

Dimension Tailoring of Quasi-2D Perovskite Films Based on Atmosphere Control Toward Enhanced Amplified Spontaneous Emission

Experimental Section

Materials: All reagents were used as received without further purification: Lead Bromide ($PbBr_2$, 99.999%, Advanced Election Technology CO., Ltd., China), Cesium Bromide (CsBr, 99.999%, Aladdin, China), Phenylethyl ammonium Bromide (PEABr, 99.9%, Xi'an e-Light New Material Co., Ltd., China), N,N-dimethylformamide (DMF, 99.9%, Super Dry, Advanced Election Technology CO., Ltd., China), Dimethyl Sulfoxide (DMSO, 99.9%, Super Dry, Advanced Election Technology CO., Ltd., China), Toluene (99.5%, Sinopharm Chemical Reagent Co., Ltd, China), Chlorobenzene (99.5%, Aladdin, China), Ethyl Acetate (99%, Aladdin, China), Isopropanol (99.5%, Maclean's, China).

Preparation of PEA_2-$Cs_{n-1}Pb_nBr_{3n+1}$ Precursor: Precursor solutions were made by dissolving $PbBr_2$ (64.4 mg), CsBr (42.6 mg), PEABr (24.5 mg) in 0.2 mL of DMF and 0.8 mL of DMSO. The precursor solutions were stirred overnight at 45 ℃ in glove box and then filtered through a 0.45 μm organic nylon filter prior to use.

Preparation of PEA_2-$Cs_{n-1}Pb_nBr_{3n+1}$ Q2D films: The substrates were cleaned step-by-step in deionized water, acetone, and ethanol through sonicating. Before spin-coating, the substrates were treated with O_2 plasma for 2 min to make the surface hydrophilic. The precursor solutions of perovskite were spin-coated onto the substrate at 4000 rpm for 45 s. By heating at 140°C, 200 μl of chlorobenzene, toluene, or ethyl acetate was volatilized into a closed space of 40×40×70 cm^3, and under this environment, the Atmosphere-Control Samples were prepared following the aforementioned procedures. Meanwhile, the control-Q2D films were prepared in air.

Characterizations: Steady-state photoluminescence (PL) emission was acquired by an F-380 spectrometer (excitation wavelength:365nm, power density: ~10mW/cm^2). Steady-state ultraviolet-visible (UV-vis) absorption was measured utilizing a Perkin Elmer Lambda 1050+ UV spectrophotometer. An Oxford Instruments Optistat-DN was used to measure temperature-dependent PL spectra in the range 80-220 K. Scanning electron microscopy (SEM) images were acquired on a Gemini SEM360 (ZEISS). Transmission electron microscopy (TEM) images were acquired on a JEM-2100 (JEOL) at 200 kV. Atomic force microscopy (AFM) images were acquired on a QUV/SPRAY (Q-panel). The photoluminescence quantum yields (PLQY) were obtained using an integrating sphere (Edinburgh Instruments, FLS920) with an excitation wavelength of 405 nm. The time-resolved photoluminescence (TRPL) decay spectra of QDs were acquired by coupling a Horiba Fluorescence spectrophotometer with a 375 nm, 45 ps pulsed laser and a time-correlated single-photon counting system. The time-resolved absorption (TA) decay spectra were measured using a 1030 nm femtosecond laser as the probing light source, which was generated through an optical parametric amplifier (OPA) to obtain a 400 nm excitation light, and using an Ultrafast Transient Absorption Spectrometer (TIME-TECH SPECTRA, TA-ONE-1) as detector. The corresponding transient absorption spectra and the lifetimes of the transient absorption were determined. X-ray diffraction (XRD) was performed using Cu Kα radiation (λ = 1.5418 Å) on an X-ray diffractometer (Bruker, D8 Focus). X-ray photoelectron spectroscopy (XPS) was recorded on a PHI 5000 Versa Probe III spectrometer using a monochromatic Al Kα radiation source (1486.6 eV). Grazing incidence wide angle X-Ray scattering (GIWAXS) data were measured by BL16B1 in Shanghai Synchrotron Radiation Facility. The in-situ PL spectra were obtained using an

optical fiber spectrometer (ATP2400, Aopu Tiancheng).

The ASE Measurements: A femtosecond laser system (343 nm, 40 fs, 66.7 μJ/pulse, 150 kHz repetition rate) is used as the laser source, and an optical fiber spectrometer is utilized for measurements. The test optical path diagram is shown as follows.

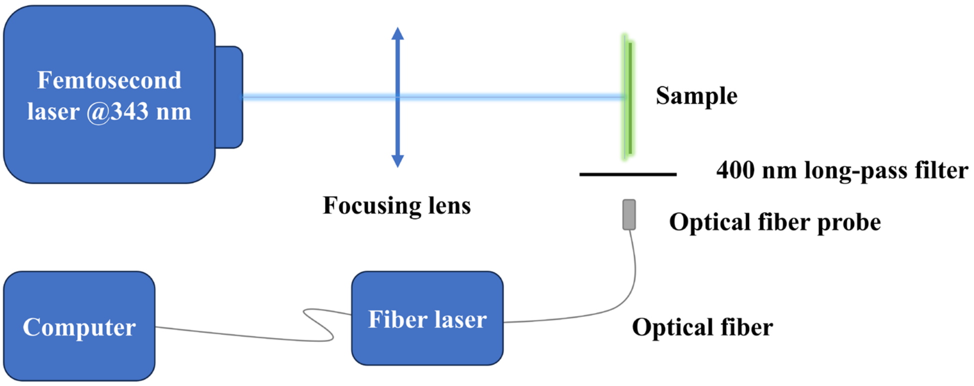

The Laser Measurements: A femtosecond laser system (343 nm, 40 fs, 66.7 μJ/pulse, 150 kHz repetition rate) is used as the laser source, two DBR with reflectance of approximately 99.5% (seven pairs of 60 nm titanium dioxide and 95 nm silicon dioxide deposited in a 5mm thick ultra-transparent glass) and 98% (four pairs of 60 nm titanium dioxide and 95 nm silicon dioxide deposited in a 5mm thick ultra-transparent glass) respectively form a resonant cavity, and an optical fiber spectrometer is utilized for measurements. The test optical path diagram is shown as follows.

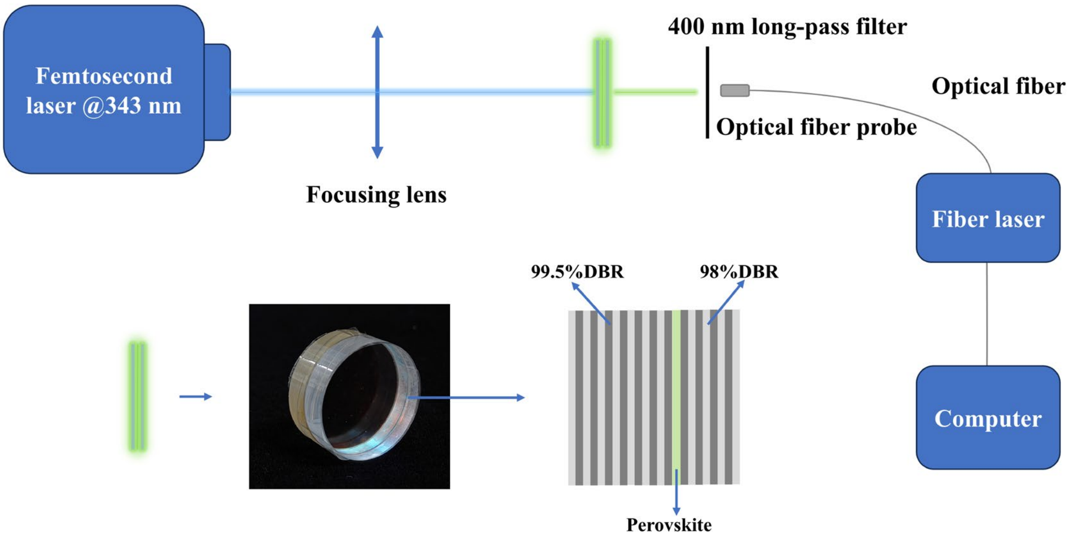

The kinetics of biexciton recombination equation (Equation S1)[1]:

$$\mathrm{I(T)} = \frac{I_0}{1+\mathrm{A}e^{-E_b/k_BT}} \tag{1}$$

I_0 is the PL intensity at 0 K; E_b is the exciton binding energy; and k_B is the Boltzmann constant.

The Phase matching condition equation (Equation S2)[2]:

$$2(\sum n_i d_i + L_{DBR1} + L_{DBR2}) = m\lambda \tag{2}$$

n_i, d_i are the effective refractive index and thickness of the material, respectively; L_{DBR1}, L_{DBR2} are respectively the thicknesses of the sedimentary layers of two different DBR; m is an integer; λ is the resonant wavelength.

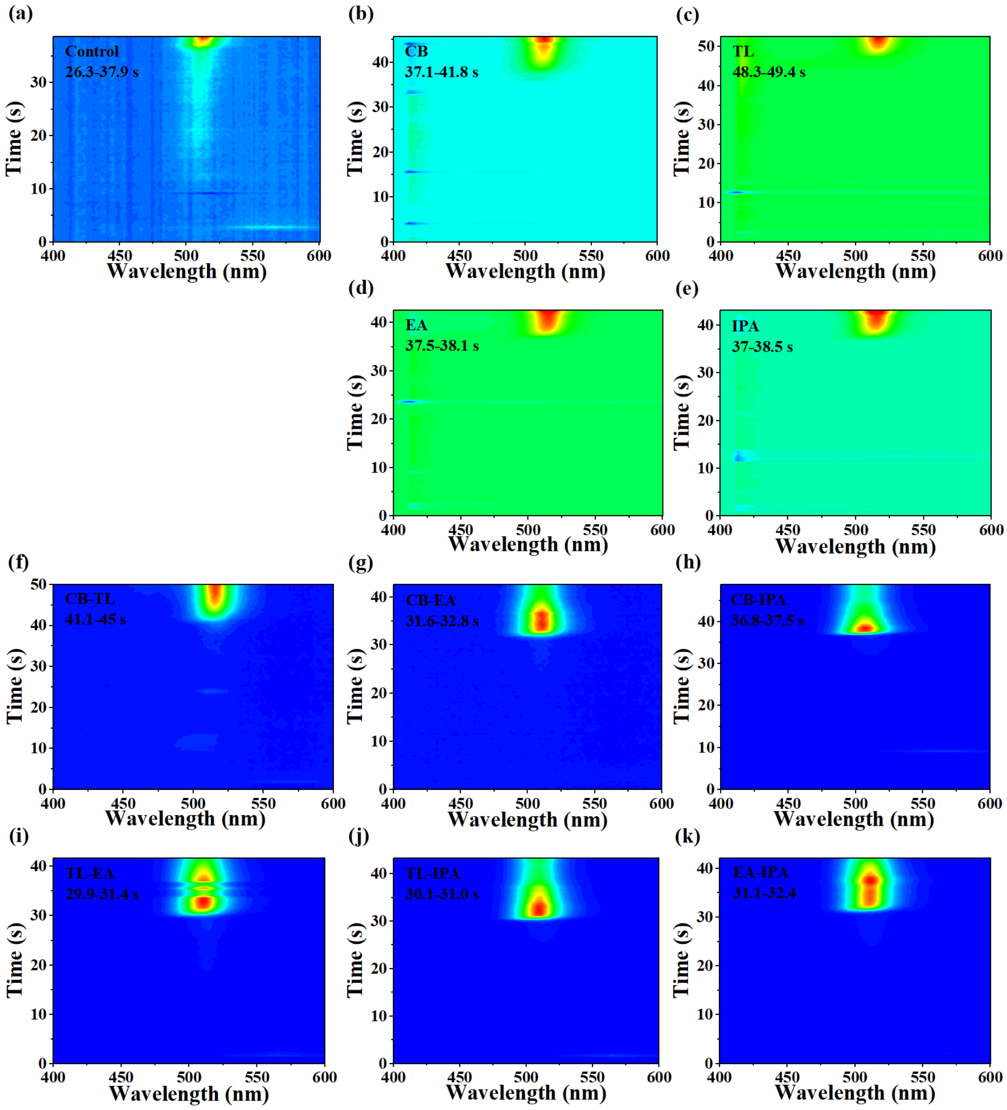

Figure S1. In-situ PL spectra of different Q2D films prepared in (a)control, (b) CB, (c) TL, (d) FA, (e) IPA, (f) CB-TL, (g) CB-EA, (h) CB-IPA, (i) TL-EA, (j) TL-IPA, (k) EA -IPA solvent atmosphere.

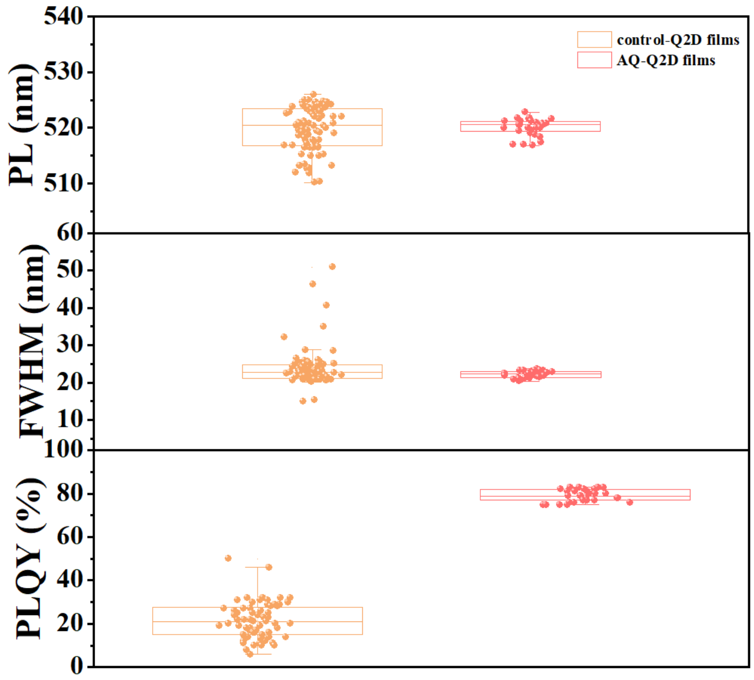

Figure S2. Comparison of batch-to-batch variation and reproducibility between control-Q2D films and AC-Q2D films.

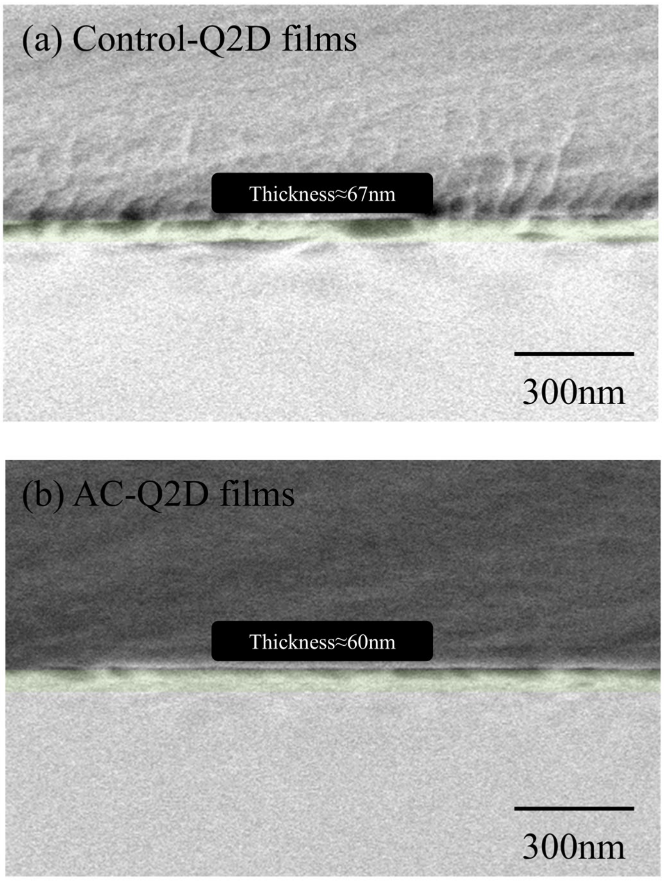

Figure S3. Cross-sectional SEM images of (a) control-Q2D films and (b) AC-Q2D films.

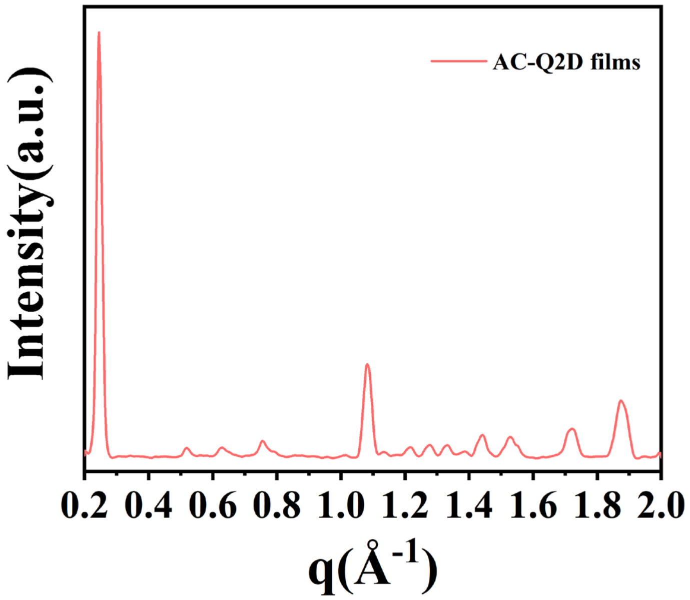

Figure S4. The integrated intensity−q relations of GIWAXS patterns of AC-Q2D films.

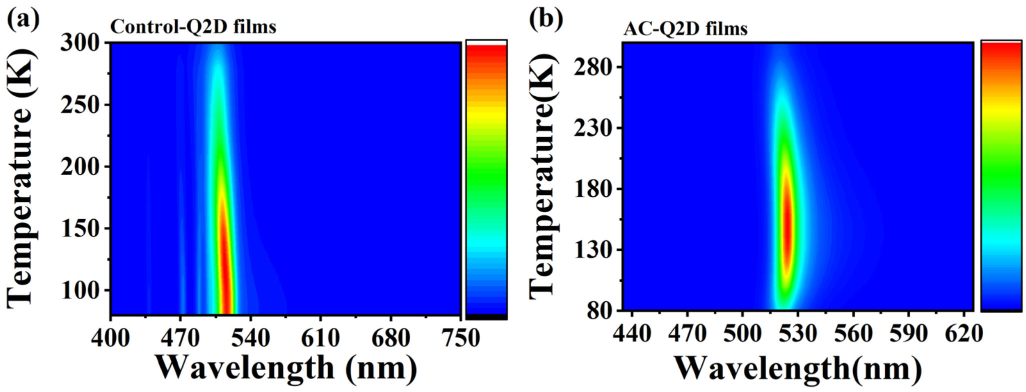

Figure S5 Pseudo color map of temperature-dependent PL spectra of (a) control-Q2D films and (b) AC-Q2D films;

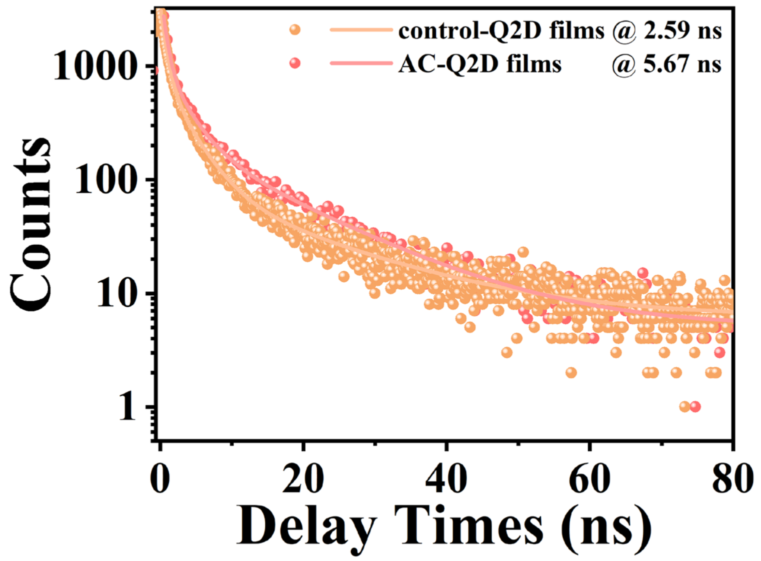

Figure S6. TRPL decay and fitting curve of the control-Q2D films and AC-Q2D films.

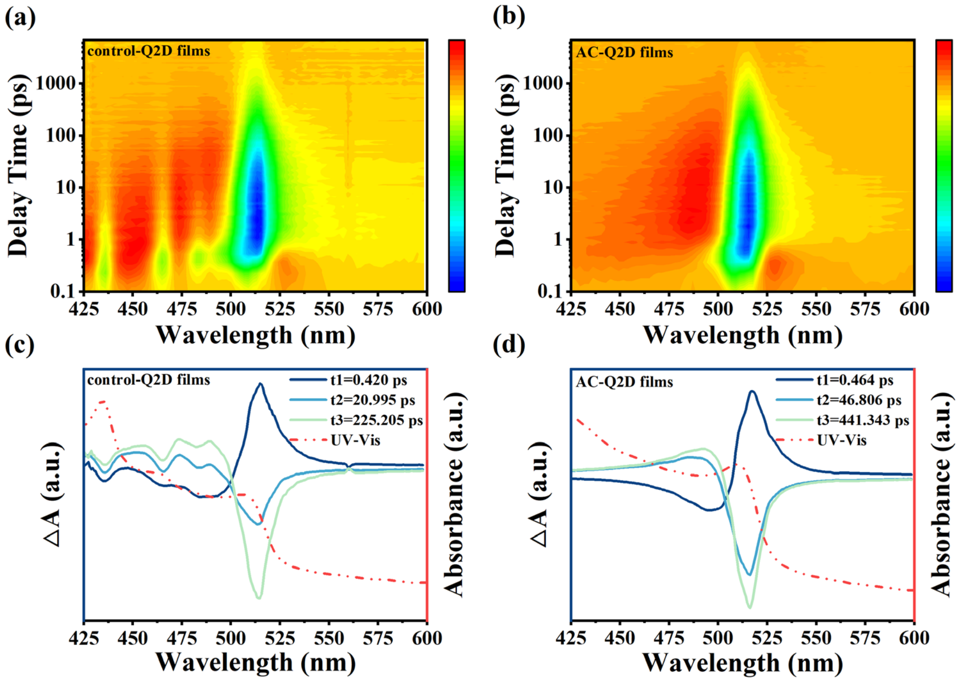

Figure S7. TA spectroscopy of (a) control-Q2D films and (b) AC-Q2D films; the SVD global fitting results for (c) control-Q2D films and (d) AC-Q2D films.

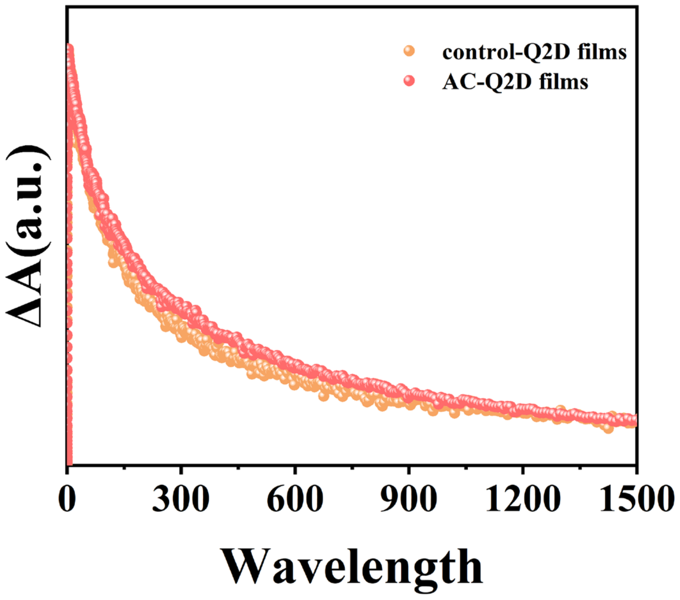

Figure S8. TA dynamics decay and fitting curve of the control-Q2D films and AC-Q2D films.

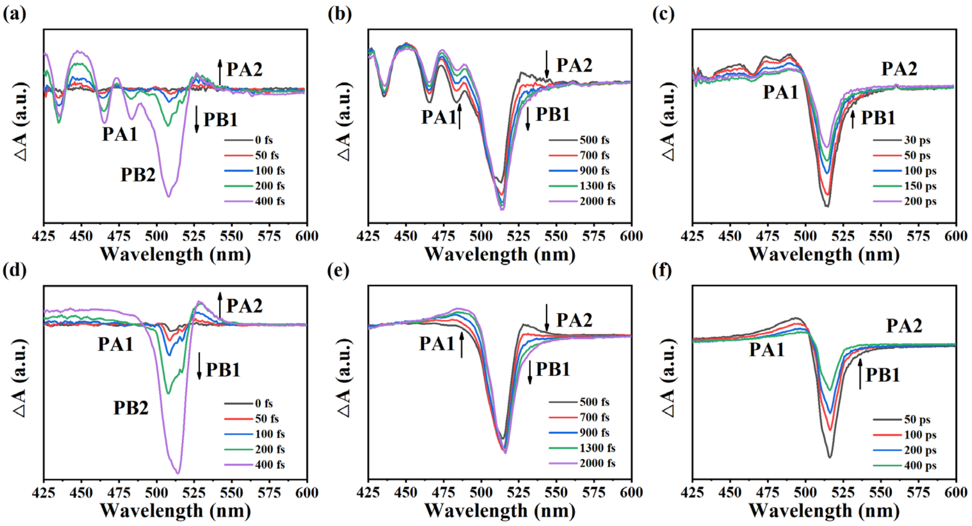

Figure S9. Time-dependent evolution of the TA spectrum of control-Q2D films in the (a) short (0–0.4 ps), (b) medium (0.5–2 ps) and (c) long (30–200 ps) time scales; time-dependent evolution of the TA spectrum of AC-Q2D films in the (d) short (0–0.4 ps), (e) medium (0.5–2 ps) and (f) long (50–400 ps) time scales.

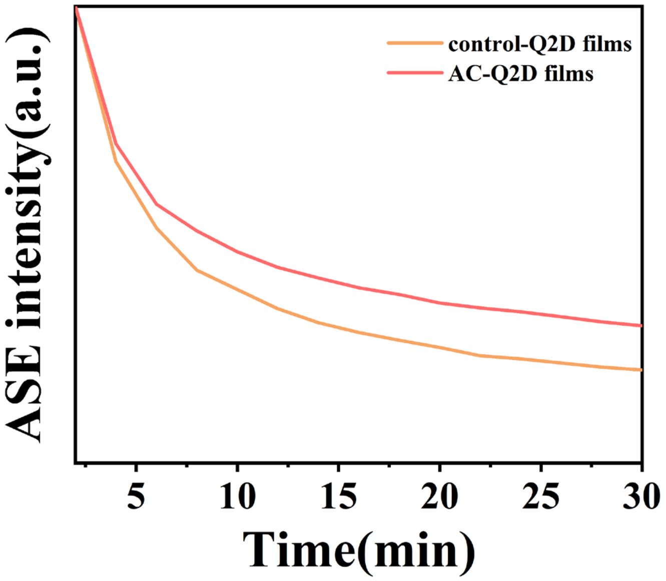

Figure S10 The ASE time stability of control-Q2D and AC-Q2D films

Table S1. Summary of TRPL fitting parameters for control-Q2D films and AC-Q2D films.

Sample Code	f_1 [%]	t_1 [ns]	f_2 [%]	t_2 [ns]	τ [ns]
contron-Q2D films	99.62	2.54	0.38	14.49	2.59
AC-Q2D films	99.09	5.44	0.91	30.95	5.67

Table S2. Summary of TA dynamics spectra fitting parameters for control-Q2D films and AC-Q2D films

Sample Code	f_1 [%]	t_1 [ps]	f_2 [%]	t_2 [ps]	T_{ave} [ps]
contron-Q2D films	15.92	95.73	84.08	748.41	644.52
AC-Q2D films	10.53	95.20	89.47	838.59	760.29

Reference

[1]. Wu, K. W.; Bera, A.; Ma, C.; Du, Y. M.; Yang, Y.; Li, L.; Wu, T., Temperature-dependent excitonic photoluminescence of hybrid organometal halide perovskite films. *Phys. Chem. Chem. Phys.* **2014,** *16*, 22476-22481.

[2]. Gao, X.; Lin, J.; Guo, X. Y.; He, G.; Zou, D. Y.; Ishii, T.; Zhang, D. Z.; Zhao, C. Y.; Zhan, H. M.; Huang, J. S.; Liu, X. Y.; Adachi, C.; Qin, C. J.; Wang, L. X., Room-Temperature Continuous-Wave Microcavity Lasers from Solution-Processed Smooth Quasi-2D Perovskite Films with Low Thresholds. *J. Phys. Chem. Lett.* **2023,** *14*, 2493-2500.
